# Older adults: panoramic view on the COVID-19 vaccination

**DOI:** 10.3934/publichealth.2021030

**Published:** 2021-05-08

**Authors:** Boris G Andryukov, Natalya N Besednova

**Affiliations:** 1 G.P. Somov Institute of Epidemiology and Microbiology, Russian Federal Service for Surveillance on Consumer Rights Protection and Human Wellbeing, 690087, Vladivostok, Russia; 2 Far Eastern Federal University (FEFU), 690091, Vladivostok, Russia

**Keywords:** older adults, COVID-19, vaccinations, aging immune systems (immunosenescence), COVID-19 vaccines, health policy

## Abstract

In December 2020, COVID-19 vaccination started in many countries, with which the world community hopes to stop the further spread of the current pandemic. More than 90% of sick and deceased patients belong to the category of older adults (65 years and older). This category of the population is most vulnerable to infectious diseases, so vaccination is the most effective preventive strategy, the need for which for older adults is indisputable. Here we briefly summarize information about age-related changes in the immune system and present current data on their impact on the formation of the immune response to vaccination. Older age is accompanied by the process of biological aging accompanied by involution of the immune system with increased susceptibility to infections and a decrease in the effect of immunization. Therefore, in the ongoing mass COVID-19 vaccination, the older adults are a growing public health concern. The authors provide an overview of the various types of COVID-19 vaccines approved for mass immunization of the population by the end of 2020, including older adults, as well as an overview of strategies and platforms to improve the effectiveness of vaccination of this population. In the final part, the authors propose for discussion a system for assessing the safety and monitoring the effectiveness of COVID-19 vaccines for the older adults.

## 1. Introduction

We live in the era of global changes in the human population's demographic characteristics. A decline in fertility and an increase in life expectancy contribute to a sharp rise in older people's proportion worldwide [1]. According to the WHO, in the early 2020, the category of individuals aged 65+ in the developed countries approached 20% of the population, and those aged 60+ constituted more than 21%. According to experts, by 2050 the total number of older adults' people in the world will increase from the current 790 million to 1.5 billion people, and the proportion of older people will increase to 29% [2].

The aging of the world's population is a severe economic and a medical-social problem, since the duration of a period of a healthy and active life lags significantly behind its duration. One of the main priorities of public health is the search for new approaches to provide older people with active old age and an optimal full and healthy life, taking into account the physiological characteristics of biological aging [3]–[5]. At the same time, the key task of current biomedical research is to identify the main processes and consequences of aging that cause loss of functions with the prospective goal of developing tools that improve their consequences [6]. One of the main consequences of aging is associated with the immune system and the associated inflammaging [5],[6].

Compared to other age groups, older adults are more prone to more frequent and prolonged infectious diseases, including viral infections, and have a high risk of reactivation of viral replication [6],[7]. Viruses activate inflammatory processes that are associated with a low level of permanent infiltration of immunocompetent cells and an increased level of some pro-inflammatory cytokines and chemokines (IL-6 and IL-8) [5],[6]. Inflammation, which is one of the critical anti-infection mechanisms at an early age, becomes a chronic pathological process after 60 years of age and a marker of aging of the immune system (immunosenescence) [4],[8].

The global COVID-19 pandemic caused by the new coronavirus SARS-CoV-2 has become a severe test for health management, healthcare policy, and healthcare economics due to the significant morbidity and mortality worldwide [9]. Epidemiological data indicate the considerable heterogeneity in the incidence rate and severity of the course and mortality from the new coronavirus infection in the population. The risk group most susceptible to COVID-19 is the older adults [9],[10].

According to the US Center for Disease Control and Prevention (CDC), at the end of December 2020, older adults Americans accounted for more than 92.45% of COVID-19 deaths in the country [10]. A similar trend was recorded earlier from the older adult age group during seasonal influenza epidemics [11].

Thus, a discussion of the impact of immune system aging is essential concerning both the current pandemic and the future threats caused by infections. This is especially true in the context of the launched mass of the population COVID-19 vaccination, which raises problems associated with the population's age heterogeneity. The experience gained from previous immunization campaigns against other viral infections [12] necessitates considering of age-related changes of the immune system, limiting vaccination effectiveness for the older adult. It is evident that drugs that are optimal for immunizing the adult population (18–60 years) will not work well enough for people over 60. This issue requires special vaccines and strategies to be designed, effectively protecting the older adults' part of the population [13]–[15].

Therefore, this review focuses on aging the immune system on immunization effectiveness in the older adult. Here we consider the COVID-19 vaccines approved for mass immunization by late 2020, with an emphasis on their use for the older adults, with a glimpse on the experience of previous antiviral vaccination campaigns. Also, we discuss the advantages and disadvantages of the currently used types of vaccines and the strategies for increasing their immunogenicity and effectiveness in the older adults group. In conclusion, we propose a system for assessing the safety and efficacy of COVID-19 vaccines for the older adults as a subject of discussion.

## 2. Immunosenescence in the older adults

Aging of the body is accompanied by a restructuring of the functionality of all systems, including the immune system. The consequence of this is a decrease in the effectiveness of immune protection, which results in an increased susceptibility to infectious and inflammatory diseases, a decrease in the reaction response to vaccination. Age-related decrease in the number of naive peripheral blood cells with a relative increase in the frequency of memory cells together with the inflammatory aging process are considered signs of immunosenescence.

Immunosenescence is a complex and polyetiological process of age-related decrease in the quantitative and qualitative parameters of the immune response, accompanied by degradation and dysfunction of many body systems, associated with the influence of external and internal factors (genetics, ecology, stress, nutrition, etc.) [16]. For example, genomic and epigenomic damage, activation of oncogenes, metabolic imbalance, and mitochondrial dysfunction are associated with stresses, the influence of which increases with age. The different contribution of external and internal factors determines the individual and population characteristics of immunosenescence, the understanding of the mechanisms of which determines the effectiveness and safety of vaccination in the older adults [7],[17].

**Figure 1.**
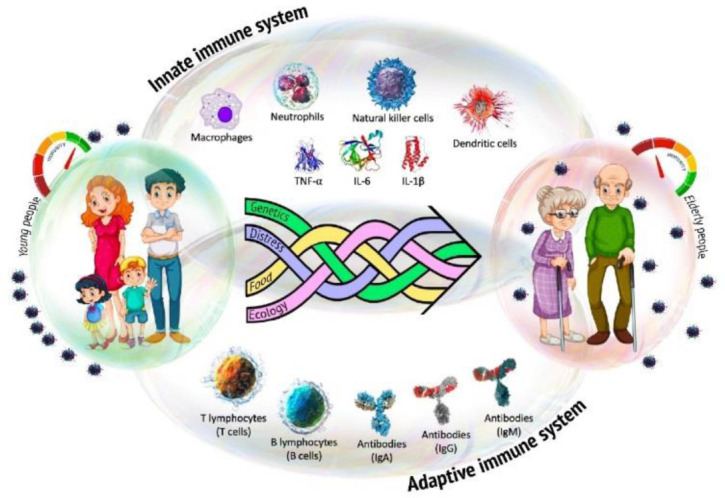
The coordinated interaction of the main components of the innate and adaptive immune systems provides an effective defense of the body against infectious agents. External and internal factors (e.g., genetics, ecology, stress, and nutrition) mediate the individual characteristics of immunosenescence.

The human immune system is a complex set of different types of immunocompetent cells, signaling molecules, tissues and organs, the complex interaction of which mediates numerous reactions, the purpose of which is to protect the body from foreign antigens [5],[7]. With age, the aging of the immune system occurs, which manifests itself in the form of a progressive attenuation of the quantitative and functional characteristics of various types of immune cells, a tendency to inflammation, an increased susceptibility to infectious diseases and a decrease in the response to immunization [5] (Figure 1).

An important sign of age-related changes in the immune system is the accumulation of senescent cells in a state of irreversible arrest of cell proliferation and reduced functionality [6]. A key feature of these cells is the formation of an aging-associated secretory phenotype (SASP), which results in the chronic induction of transcription and the secretion of many pro-inflammatory cytokines, chemokines, growth factors, and matrix metalloproteinases [18],[23]. With an increase in the general inflammatory status, due to increased activation of cytokine secretion, a weakened immune response to vaccination in older adults is associated, which leads to obvious problems in the development of a vaccine in this age group of the population [23].

The effective defense of the body against infectious agents is mediated by the coordinated interaction of the main components of the innate and adaptive immune systems formed during human development. The division of this tandem is conditional. The evolutionarily more ancient innate immune system provides a fast and effective immune response. It provides the non-specific recognition of molecular patterns of invading pathogenic organisms using the pattern recognition receptors (PRR) and signaling molecules [18],[19].

The received signals mediate the long-term antigen-specific memory of the adaptive immune system associated with the effector potential of its main components, T- and B-lymphocytes (T- and B-cells) [6],[19],[20]. These processes result in a significant and rapid secretion of pro-inflammatory molecules (cytokines, chemokines, and interferons), stimulating other cellular reactions and producing specific antibodies that protect the host organism [21]–[23].

However, as the body ages, this multilevel system fails. The age-related decrease in immunity is characterized by heterogeneous dysfunction of innate and adaptive immunity components. It is believed that the process of immunosenescence is individual and is the primary explanation for the increased risk of having infectious and systemic diseases at old age against the background of progressive chronic inflammation [22]–[24] (Figure 1).

Age-related signs of aging are associated with remodeling of the ***innate immune system***, which is the first line of defense of the body, include a wide range of suppressive processes, reflecting the general picture of a deep dysregulation of its functions. They are associated with progressive dysfunction of its main cellular components, which are of key importance in early immune responses and antigen presentation: macrophages, neutrophils, natural killer (NKs) cells and dendritic cells (DCs) [23],[25]–[27]. Age-related functional dysfunctions of these cell types are mediated by changes in receptor expression, a decrease in cytokine production and the activity of signaling molecules [22]–[27].

In *macrophages*, age-related changes are primarily manifested as a decrease in the expression of surface molecules, such as major histocompatibility complex II (MHC-II) and toll-like receptors (TLRs), and in the efficiency of antigen presentation and interleukin production [28]–[30]. In addition, in the older adults, the phagocytic ability of macrophages is reduced by inhibition of the secretion of chemokines MIP-1α, MIP-1β, MIP-2 and eotaxin (member of the PF-4 family of chemokines), as well as a decrease in phosphatidylinositol-2-kinase protein kinase B (PI3K-AKT) and cyclic GMP- AMP-synthase stimulator of interferon gene signaling (cGAS-STING) [23].

The activation and control of the immune response is highly dependent on the function of DCs, being specialized antigen-presenting components of innate immunity, capture and process antigens for T-cells presentation, mediating adaptive immunity's effectiveness [31],[32]. Numerous DCs receptors (PRR, TLR, C-type lectin, etc.) perceive both external antigens and internal signals and hazard molecules (e.g., autoantigens), which play an essential role in maintaining immune homeostasis and preventing autoimmune reactions [31]–[33]. As the body ages, there is a decrease in the number of receptors and a dysfunction of the initiation of an adaptive immune response [8],[32],[34]–[36]. It was found that coronaviruses are infects DCs, which leads to the induction of the secretion of proinflammatory cytokines and a further damaging response [29]. Recent studies, carried out in October 2020, revealed impaired DC maturation and subsequent T-cell responses in response to SARS-CoV-2 infection [29],[36].

*Neutrophils* (or polymorphonuclear neutrophil leukocyte, PNL) are the first cells to migrate to the inflammation site, where they phagocytose and kill pathogens. In recent years, it became known that these cells are actively involved in regulating the immune system's effector link, synthesizing and secreting a wide range of cytokines and chemokines [37],[38]. Besides, neutrophils' phenotypic plasticity allows them to express functional MHC-II molecules and, possibly, functional T-cell receptors, which mediates deep functional connections of PNL with the main components of adaptive immunity [39]–[41].

**Table 1.** The most pronounced signs and consequences of aging of the immune system (immunosenescence) in COVID-19.

Process markers	Signs of Aging	Implications for the Body with COVID-19	Refs
Natural killer (NK) cells	Decline in cytotoxicity and cytokine (chemokine) generations	Low NK activity is associated with a higher risk of respiratory infections.	[23],[25]–[27]
Neutrophils, monocytes, macrophages, and dendritic cells (DC)	Decrease in number and lower expression of surface molecules (MHC-II, TLRs). Reduced number and functionality of DCs	Poor priming of T-cells, decreased phagocytic and chemotaxis activity, leads to impaired antigen presentation and activation of adaptive immune responses. Disturbed processing and presentation of phagocytes and antigens.	[8],[31]–[33],[34]–[36]
Pro-inflammatory markers: cytokines (TNF-α, IL-6, and IL-1β)	Increased secretion	Release of pro-inflammatory mediators of inflammation (cytokines), which can develop into ‘cytokine storm’.	[5],[30],[42],[43]
T- and B-cells, their subpopulations and interaction with macrophages and dendritic cells (DCs)	Inflammageing of the immunocompetent cells and their precursors. Impaired cell-mediated response	Decreased functionality of the innate and adaptive immune systems, impaired response to vaccination. Lack of cross-reactive T-cells that react with SARS-CoV-2.	[3],[6],[7],[28],[44]–[46]
Antibodies, their products and functionality	Decreased rates of seroconversion and seroprotection	Synthesized antibodies do not perform protective function. Risk of infection-induced pneumonia.	[14],[15],[47]–[50]

Note: MHC-II—major histocompatibility complex II; TLRs—toll like receptors; TNF-α—tumor necrosis factor-α; IL—interleukin.

An example of the most pronounced processes of immune aging are age-related changes in the phenotype and subpopulations, as well as a decrease in the activity of *natural killer (NKs) cells* [23],[25],[26]. These cells are designed to recognize as well as direct and rapid MHC-independent destruction of infected and cancer cells by releasing perforin and granzymes [24]–[26]. With age, NKs show a significant decrease in their functionality, including cytotoxicity and cytokine production [23],[26], which may alter the expected response to vaccines in the older adults [23],[26],[27] (Table 1).

One of the pathogenetic mechanisms responsible for the severe course and poor outcome in COVID-19 is associated with viral stimulation of the innate immune system. This process is accompanied by the release of pro-inflammatory and inflammatory mediators (cytokines), developing into a ‘cytokine storm’. It is an inflammatory response of organisms, with cytokines in the blood increasing sharply, which causes the immunity to attack cells and tissues of own organism. A consequence of this response can be the destruction of tissues and organs and, as a result, the death of the organism [30],[42],[43].

In older adults, severe inflammatory reactions cause a sharp deterioration in the condition or death. In the absence of immunosuppressive treatment, they can cause irreversible systemic pathological processes in the tissues of the lungs, heart, and brain and dysfunction of the adaptive immune system [51].

The effect of aging ***on adaptive immunity*** is mediated by reducing the diversity and functionality of its main components, T- and B-lymphocytes (T- and B-cells) [28],[44]. It was found that these disorders are associated with involution of the thymus and a decrease in the number of naive T- and B-cells and an increase in the number of effector and memory cells. At the same time, there is an accumulation of terminally differentiated, aging and depleted effector T-cells and a decrease in the number of naive T-cells and their functional defects [7],[28],[44]. This leads to a violation of the proliferative ability, a decrease in the functionality of the T-cell link and the interaction between mature and naive cells, as well as a change in the production of cytokines [6],[7],[28].

With aging of the immune system, the pool of B-cells also qualitatively changes due to a decrease in the number of naive B-cells and the functionality of mature B-lymphocytes. This mediates a decrease in the production of specific antibodies during the immune response, while increasing the induction of the formation of autoantibodies, which is often observed in old age [7],[52],[53].

For many types of immunocompetent cells, age-related metabolic changes have been described, the significance of which for age-related dysfunctions of the immune system has recently been recognized more and more. In particular, the chronic subclinical inflammatory status, which is observed in the older adults, can be explained, inter alia, by the impaired production of cytokines by the cells of innate and adaptive immunity, as well as adipocytes, fibroblasts and other types of cells [7],[44],[52]–[54].

As has been established by modern imaging methods, the proportion of the CD8+ subpopulation in the T-cell population increases with aging. These cell subtypes are characterized by the lack of co-stimulating adhesion molecules CD28 (CD28–). The antigen presentation without co-stimulation leads to T-cell anergy development [53]. The predominance of the CD8+ CD28– cell subpopulation in the peripheral pool of CD8 (more than 50%) is currently considered a biomarker of aging of the T-link in the adaptive immune system [53],[54]. Age-related changes in the immune system have a direct impact on the immune responses mediated by vaccination.

In a normal response to vaccination, immunocompetent cells of the innate immune system recognize vaccine antigens at the injection site, mediating a local inflammatory response. Antigens are taken up by phagocytes and presented to T-cells, whose optimal functionality is critical for the production of specific antibodies by B-cells, the concentration of which in serum is important in determining the effectiveness of a vaccine [7],[28],[44],[54]. Thus, an adequate cell-mediated immune response is essential for vaccination.

Thus, a recent study by McElhaney et al. [52], studying the effects of immune aging in older adults' patients with a viral infection (influenza), concluded a change in the cell-mediated response. Seroconversion and seroprotection rates were significantly higher in younger volunteers than in older people [52].

When identifying the causes of high mortality from COVID-19 and a more severe course of infection in the older adults' population, Saletti et al. [55] concluded that a possible explanation for this phenomenon is the almost complete absence of cross-reactive T-cells that react with SARS-CoV-2 in patients of this age group [55].

Thus, dysfunctions in the T-cell link and loss of CD28 expression are key factors in immune aging. This alone is sufficient to explain the decrease in antibody titer after vaccination in older adults, which cannot be attributed solely to defects in B-cell function.

B-lymphocytes are of crucial importance for humoral immunity [56]–[59]. B-cells' most critical functions in mediating the immune response in infections are related to their ability to differentiate into plasma cells that produce protective antibodies [57],[59]. Distinct subpopulations of B-cells mediate different types of immune responses. After meeting with the antigen, mature B-cells mediate the secretion of a diverse set of specific antibodies (IgA, IgM, IgG, IgE). Also, B-cells play an essential role in the immune system through antigen presentation and cytokine secretion [56],[58].

However, the manifestation of B-cell dysfunction during aging of the immune system has long remained unclear [60],[61]. In recent studies, it has been found that with aging of the body, this clan of immunocompetent cells, as well as their precursors, is also subject to involutional changes, which is of key importance in the SARS-CoV-2 infection and for effectiveness of COVID-19 vaccination [61]–[63].

Thus, the works of Hartley et al. [64] и Cañete & Vinuesa [65] it has been shown that the effectiveness of modern COVID-19 vaccines largely depends on the induction of memory B-cells (and long-lived plasma cells), which provide continuous synthesis of high-affinity antibodies [64],[65]. In another study, Sosa-Hernández et al. [66] have found that the severe course of the new coronavirus infection is accompanied by the emergence of immature and incompletely differentiated clusters in a subpopulation of B-lymphocytes, which can potentially become a prognostic clinical biomarker indicating a disturbance of antibody production [66].

Similar processes are observed during the aging of the immune system. In recent years, the discovery of age-associated B-cells involved in the pathogenesis of autoimmune diseases in the older adults and quantitative and qualitative changes in the production of specific antibodies (decreased titers and lower affinity for antigens) have attracted considerable attention [44],[56],[67].

For example, Park et al. [68], Studying the immunological basis of the age-related lower efficacy of the pneumococcal polysaccharide vaccine (PPV23), it was found that after the administration of PPV23 in the older adults, there was a decrease in the number and deficient IgM memory B-cells and the production of high-affinity antibodies [68].

The predominant secretion of pro-inflammatory cytokines accompanies these processes. The elevated levels of TNF, IL-6, and IL-1β mediate the state of chronic inflammation that develops in most older adults, as well as an increased risk of other adverse health effects [42],[63]. In addition, the immune response is negatively affected by the presence of chronic diseases, as well as excess adipose tissue (obesity), which are widespread among the older adults [28],[52]–[54].

Thus, a decrease in the functionality of the immune system is likely to lead to a decrease in vaccination efficiency in older adults. In addition, the impairment of the cell-mediated immune response casts doubts on the adequacy of traditional antibody testing in assessing the effectiveness of COVD-19 vaccines in this age group of the population. Moreover, over-activation of immune responses poses a risk of unwanted complications associated with the general pro-inflammatory status in older adults, raising concerns about the safety of vaccines in the concept of immunosenescence.

## 3. COVID-19 vaccination in the concept of immune aging

In the public health system, vaccination is one of the most effective measures to prevent the spread of infections. The critical principle of vaccination is the stimulation of the body's immune response and the formation of population immunity in the population to protect against infectious or other diseases [3]. In the history of implementing international and national immunization programs, examples of the almost complete elimination of certain infections (smallpox, poliomyelitis, and others) are known [3],[69]–[74].

In December 2020, the mass COVID-19 vaccination, a contagious disease caused by a previously unknown coronavirus, was launched in many countries [75]. Despite the high contagiousness of SARS-CoV-2, the age spectrum of patients is unevenly presented, the bulk of COVID-19 patients are patients over 65 years old. Accordingly, the risk of mortality from a new coronavirus infection increases sharply in older adults [1],[75]. Therefore, the only hope for halting the spread of the pandemic is vaccination.

The main goal of COVID-19 vaccination is to prevent this serious infection, reduce the risk of complications and death, which is especially important for high-risk groups, especially older adults. Expanding vaccination coverage in this age group, in general, will increase the effectiveness of the entire immunization campaign in the population by reducing the foci of spread and circulation of the pathogen and, consequently, reducing the socio-economic impact of the pandemic [70],[73],[75].

One of the consequences of immunosenescence in the older adults is heterogeneity and weakening of the immune response to vaccination, which may be necessary in assessing the effectiveness of vaccination in this population. Thus, older people are the most vulnerable group of the launched prevention campaign population.

For example, in the already mentioned study, McElhaney et al. [54] assessed the aging immune system's response to influenza vaccination. It was found that in the older adults, the ability to generate high-affinity antibodies decreases, and the administration of the vaccine caused only a weak response of the T-cell link of the adaptive immune response [54].

In this regard, the evaluation of the results of clinical trials of vaccines in this age group of the population should not be limited only to the conclusion about their safety (this is an absolute and necessary condition for their use), but should include the characteristics of the immune response [3],[70],[72]. Conclusions about the feasibility of using vaccines for the older adults must be based on the understanding that success is limited by the aging of adaptive immunity, which is a critical factor in vaccine development [3],[12],[13],[25].

Fast forward to early 2020, the world's major pharmaceutical companies, using traditional approaches and financial support from their governments, began an unprecedented race to develop and test safe and effective candidates for COVID-19 vaccines under conditions of extreme time pressure. As a result, by late 2020, more than 180 various types of drugs were announced to protect against COVID-19 by inducing the production of specific antibodies against SARS-CoV-2 [75],[76]. By that time, many leading drug manufacturers had successfully carried out clinical trials and got the required official approvals for their vaccines to launch the population's mass immunization [14],[15],[49].

The most effective of the vaccines will become evident with time. Below, we list the most common new and traditional platforms to develop the most advanced COVID-19 vaccines for mainstream use (Table 2).

**Table 2.** Most common platforms used to develop COVID-19 vaccines.

Vaccine types	Design and operation principle	Examples of leading vaccines	Refs
Inactivated or attenuated coronavirus vaccines	Designed on the basis of an attenuated or weakened form of SARS-CoV-2 that is unable to cause infection, but can induces the production of antibodies	CoronaVac (Sinovac Biotech); BBIBP-CorV (Sinopharm-Beijing); Sinopharm-Wuhan (all – China)	[14],[15],[49]
Protein-based vaccines	Safe fragments of proteins or protein shells that mimic the COVID-19 virus to induce the immune response	EpiVacCorona (Vector, Russia); NVX-CoV2373 (Novavax, US)	[77]–[80]
Viral vector vaccines	Vaccines based on completely different viruses (e.g., adenoviruses) with a small alien gene inserted, which is a region of the SARS-CoV-2 genome. As part of safe viruses (vectors), this gene enters a host cell and produces coronavirus proteins to safely induce the immune response.	Sputnik V (RF); Convidecia (CanSino Biologics China); Ad26.COV2.S (Johnson & Johnson US, Germany); Oxford-AstraZeneca (UK)	[81]–[84]
RNA and DNA vaccines (Genetic Vaccines)	DNA vaccines contain a circular DNA molecule (plasmid), which encodes a viral protein. RNA vaccines contain messenger RNA (mRNA). This molecule is a “matrix” for the subsequent synthesis of a viral protein, to which an immune response is then expected.	Comirnaty (Pfizer-BioNTech, US); мРНК-1273 (Moderna, US)	[85]–[87]

The principle of operation of the proposed types of vaccines is based on various mechanisms of generating an immune response in the body. Each platform has its advantages and disadvantages [79],[81],[84]–[86] (Table 3).

**Table 3.** Advantages and disadvantages used of the types of COVID-19 vaccines.

Types of vaccines	Advantages	Disadvantages	Refs
Inactivated or attenuated coronavirus vaccines	*Attenuated vaccines:* Immunity from this vaccine lasts for the longest period, which is especially important in the case of COVID-19, because coronavirus infections do not always induce long-term immunity (antibody) responses. *Inactivated vaccines:* Inactivated vaccines are safer than attenuated ones, as they do not contain “live” virus that can mutate	*Attenuated vaccines:* Due to the mutation of SARS-CoV-2, the process of creating a “live” vaccine is largely unpredictable. There is always a risk that the weakened virus “regains its strength” and “learns” to cause the disease. *Inactivated vaccines:* - cause an overly weak immune response (adjuvants required); - the formed immunity is not as persistent as that from attenuated vaccines	[88],[89] [90],[91]
Protein-based vaccines	This vaccine is safe for human organism, and, thus, can be quickly tested and used for mass vaccinations. In combination with an adjuvant, it enhances the immune response.	Obtaining a quantity of viral protein enough to make a vaccine is very difficult. Moreover, immunity to such vaccines is unstable, necessitating adjuvants' use.	[79],[80],[92]
Viral vector vaccines	Vector vaccines form the same strong immunity as attenuated, but they will not be able to mutate.	Vector vaccines based on adenoviruses are not well understood. Attempts to develop vaccines against cancer, HIV, influenza, and Ebola have already been made, but so far, none have been approved for humans. Pre-existing immunity against vectors can alter the subsequent immune response to the vector antigen	[93]–[95]
RNA and DNA (genetic) vaccines	*DNA vaccines:* immunity is as long-term as that of “live” vaccines, but without the risk of mutation and infection. *RNA vaccines:* lipid particles with mRNA can elicit an immune response. Immunity from RNA vaccines is expected to develop earlier and last longer. In addition, mRNA can be created relatively quickly and cost-effectively using special synthesizers. In theory, this technology is the safest.	*DNA vaccines:* - poorly understood; - only one vaccine of this type is used currently, the Zika virus vaccine for horses; - no DNA vaccine has been approved for humans to date. *RNA vaccines:* - an absolutely new vaccine type that is poorly understood; - the behavior of RNA vaccines in the human body has not yet been characterized; - there is a complete lack of experience in using vaccines of this type.	[96]–[98]

One of the most well-known technological platforms used to create COVID-19 vaccines is highly attenuated (***attenuated coronavirus vaccines***) strains SARS-CoV-2. The mechanism for obtaining these drugs is based on multiple coronavirus passages in animals. As a result, the virus mutates, adapts to a new host, and weakens in relation to a person, but remains capable of causing an immune response in him [99],[100].

The major challenge in creating such a live vaccine is associated with the mutation process's unpredictability and the risk of re-gain of pathogenic properties by the weakened virus after vaccination [101],[102]. The immunity from such a vaccine lasts for the most extended period, which is especially important in COVID-19, but it shows some specifics in older adults' people.

Roukens et al. [103] studied the immune response in older adults (60–81 years, N = 28) after primary immunization with a live yellow fever vaccine (YF-17D). This age group had a delayed antibody response with a higher viremia level. The authors concluded that the weaker and more delayed immune response to the yellow fever vaccine allows the weakened virus to replicate and cause higher viremia levels [103], which can cause fatal side effects from vaccinations.

Similar studies have shown that after immunization with vaccines of this type, the maximum titer of neutralizing antibodies in the older adults is delayed by about a week, which is associated with a slowdown in the induction of the immune response's effector phase [47],[104].

Vaccines containing inactivated strains of SARS-CoV-2 are created on the same platform of whole-pathogenic viruses. For ***inactivation coronavirus vaccines***, viruses are heated or exposed to ionizing radiation or disinfectants [105],[106]. Viral proteins change spatial configuration but retain their chemical composition and immunogenic properties. The major problem with these vaccines is that the immune response formed is too weak, which requires repeated vaccinations or the use of adjuvants (enhancers of immune responses) [106].

Developers of both live and inactivated vaccines against various viral infections (hepatitis B, influenza, Japanese encephalitis, etc.) in the 20th and 21st centuries solved their insufficient immunogenicity and effectiveness [47],[107]. Nevertheless, older adults show a significantly lower production of specific antibodies than younger adults or an inability to maintain the protective antibody titers [47],[107]–[109].

For example, in a recent monocentric study, Wagner et al. [107] showed that after primary immunization against Japanese encephalitis, about 50% of people over 60 (61–78 years, N = 30), compared to the number of young participants (18–30 years, N = 30), did not produce the levels of antibody titer required for the protective response [107].

Among the probable mechanisms of insufficient immune response in the older adults after primary immunization with inactivated vaccines, the authors indicate the age-related decrease in the functionality of the T-cell link of immunity (CD8 and CD4). This leads to reduced production and a rapid decrease in antibody titers [103],[104],[107]. This will probably require regular booster vaccinations to ensure the protection of this population.

The significant success in the creation of this type COVID-19 vaccines has been achieved by Chinese developers, whose products have successfully passed clinical trials and are admitted for mass immunization at the end of 2020.

For example, CoronaVac, based on inactivated coronavirus, developed by the private company Sinovac Biotech, has shown promising results and no side effects in clinical trials conducted in Brazil, Indonesia, and Turkey. The vaccination campaigns with this vaccine are currently being carried out in China and Indonesia [75],[77],[86].

Furthermore, another Chinese vaccine of a similar type, BBIBP-CorV (Sinopharm-Beijing), has been successfully tested in the United Arab Emirates, Peru, and Morocco, which allowed receiving permission for mass immunization of specific population groups in China in November 2020 [15].

***Protein vaccines*** can be designed based on the structural proteins of SARS-CoV-2 (spike (S), membrane (M), envelope (E) embedded in the surface envelope of the virus, as well as the N nucleocapsid protein). Unlike the S-protein and its epitopes, the M and E proteins are less immunogenic and have not yet been used to create COVID-19 vaccines. However, M- and E-proteins showed higher sequence identity among SARS-CoV, MERS-CoV, and SARS-CoV-2, suggesting their high potential for cross-reactivity when included in a vaccine [9].

Besides, this coronavirus encodes 16 more non-structural (nsp1-16) and 9 additional proteins that can potentially be used to create vaccines and be targets for an immune response [9],[12].

The clinical trial threshold has been successfully crossed by EpiVacCorona preparations (NPO Vector, Russia) and NVX-CoV2373 (Novavax, US). At the same time, EpiVacCorona is designed based on three different chemically synthesized peptide antigens of the protein S of the SARS-CoV-2 virus, conjugated with a carrier protein and an adjuvant of aluminum hydroxide [75],[77]–[80].

NVX-CoV2373 is a recombinant nanoparticle vaccine constructed from SARS-CoV-2 full-length glycoprotein S, containing an adjuvant Matrix-M1 [79]. Simultaneously, the American company reached an agreement to sell tens of millions of vaccine doses to Australia, New Zealand, and India [79],[86].

The weak point of protein vaccines is the difficulty of obtaining a sufficient number of viral proteins for vaccine production and the need to introduce adjuvants into the composition to increase the immune response [80],[92].

One of the most popular modern types is vector vaccines produced based on safe carrier viruses (viral vectors). According to the WHO report of December 23, 2020, four strong players have achieved the tremendous success on the platform of vector viral COVID-19 vaccines: the Gamaleya National Center of Epidemiology and Microbiology (Sputnik V), the Chinese company CanSino Biologics (Convidecia), the American company Johnson & Johnson (Ad26.COV2.S), and the Anglo-Swedish pharmaceutical company (Oxford-AstraZeneca) [81]–[84].

This technology attracts drug manufacturers because a viral vector can infect human cells only once and cannot reproduce in the body, making this type of vaccine safe. Adenoviruses are most promising to be used as carrier viruses to transfer foreign genes [86],[87]. For this reason, when developing their vaccines against SARS-CoV-2, all of the above-listed leading drug manufacturers used these carrier viruses as a vector [86],[93],[94].

One of these viruses' main advantages is their natural mechanism of interaction with human cells, which allows them to provide sufficient long-term antigen expression, which effectively activates both innate and adaptive immune responses [87],[94],[95].

Considering the peculiarities of the aging immune system's dysfunction, the main disadvantage of adenoviruses should be highlighted. It is associated with their pro-inflammatory properties, which, on the one hand, causes a strong immune response, and on the other hand, it can mediate unpleasant complications after vaccination in the older adults [86],[87],[95].

Another unpleasant aspect is associated with the fact that most adenoviruses are ordinary human highly immunogenic respiratory pathogens. Most older people have become infected with adenoviruses during their lifetime, have developed and retained virus-neutralizing antibodies to them, which are a significant obstacle to the successful use of these viruses as a vector of vaccines in the human population [110]–[113].

Thus, the studies of Farina et al. [110] and Vogels et al. [112] would show that in the population of certain regions, from 45 to 80% of adults carry adenovirus-neutralizing antibodies in various titers [110],[112]. Even in low titers, these antibodies reduce the uptake of adenoviral vectors by cells, including immune antigen-presenting cells, which can reduce vaccines' effectiveness or lead to unpredictable immune responses, especially in older adults' patients [112]. One of the possible solutions to this problem is associated with the modern strategy of using adeno-associated viral vectors, to which there is no immune response that reduces the effectiveness of vaccines [113].

***Genetic vaccines*** can be of two subtypes: DNA and RNA vaccines.

DNA vaccines contain a circular DNA molecule (plasmid). This molecule contains the “instructions” for making a viral protein. After entering a vaccinated person's cells, circular DNA becomes part of his genome. The cells of the host organism receive a new “instruction”, according to which they begin to produce viral antigen proteins, to which an immune response will be formed. Sometimes, to deliver the target DNA molecule into the cell, it is inserted into a safe carrier virus genome used as a “syringe”. Unlike vector vaccines, only the envelope is used from the safe virus [114]–[117].

These vaccines have the same advantages as vector vaccines: persistent immunity and inherent disadvantages. Since SARS-CoV-2 is not taken as a “carrier virus” for the plasmid, there is no danger that the weakened virus will mutate and cause disease again [117],[118].

The problem with these vaccines is that they are poorly understood. Only one vaccine of this type has been developed and used—against Zika for horses [114],[119].

The technology-based on using messenger RNA to create vaccines, proposed in the 1990s, is more optimized than traditional platforms. It significantly reduces many of the stages in vaccine development. RNA vaccines contain a viral molecule referred to as messenger RNA (mRNA), similar to DNA structure. This molecule is a template from which a viral protein is synthesized directly. B-cell genome of mRNA is not inserted.

Such a vaccine's mechanism of action is to transport the mRNA inside the lipid nanoparticle into the body's cells. Further, the lipid component is incorporated into the target cell membrane. Once in the cell, the viral molecule becomes a template for the synthesis of viral antigen proteins that cause immunization [115]. The benefits of this vaccine are the same as those of DNA analogs. At the same time, lipid nanoparticles themselves can induce an immune response. It is assumed that this dual immunogenic effect may induce immunity earlier and last longer [114],[117],[120].

The main problem with these vaccines arises from the fact that this is a novel technology. No vaccines of this type exist to date [119],[120]. For example, the American company Moderna has tested several mRNA vaccines against some causative agents (such as cytomegalovirus, chikungunya, and Zika viruses) over the past few years but has not offered any of them on the market. In January 2020, the manufacturer began developing a coronavirus vaccine dubbed mRNA-1273. Already on December 18, the company received official permission for the emergency use of a new vaccine with a declared efficiency of 94.5% [121].

Another American company BioNTech (together with the pharmaceutical giant Pfizer with a century and a half history) used the same principle to develop a vaccine Comirnaty (Tozinameran or BNT162b2) and is approved for immunizing people over 18 years of age [85],[87]. The vaccine contains the mRNA of the SARS-CoV-2 virus, which encodes the synthesis of the coronavirus spike protein, which triggers an immune system response [85]. BNT162b2 was the first of its kind to receive an emergency use approval from WHO as “meeting all essential safety and efficacy parameters”. Pfizer's statement of 94% vaccine effectiveness for people over 65 is encouraging and means that the risk of infection in this age group will be reduced by 20 times [75],[76].

The problems of using the developed COVID-19 vaccines are associated with the expensive organization of the cold chain for its storage (−70°C), which, given the costly production, raises some doubts about the future of this RNA technology.

So far, all the developers give optimistic forecasts about their drugs' incredible effectiveness (85–95%), which exceed the most optimistic forecasts (the most effective influenza vaccine has an efficiency of about 50%). It is hoped that all the declared vaccines are of different types and, possibly, will not so much compete as complement each other, which will be crucial for mass immunization. Their safety and efficacy for older adults are critical to the success of global immunization.

Moreover, despite the great social importance of COVID-19 vaccines, there remains a sense of uncertainty due to concerns that this competition is determined by other rules: vaccines were licensed before they were proven to be effective and safe for the general population, in particular, for older adults, who bears the brunt of the current pandemic. This category of people is most prone to severe infection but was not included or underrepresented in clinical trials. In addition, Soiza et al. [122] acknowledge that the effectiveness of the proposed vaccines has generally not been adequately studied for older patients, since the evaluation of the studies excluded older adults over 85 years of age and with multiple comorbidities [122].

The lack of complete information also arouses uncertainty feeling. Therefore, to achieve the absolute confidence in the campaign and a level of immunization required for herd immunity (>60%), a special strategy should be designed for monitoring the effectiveness of COVID-19 vaccination in the older adult's group of the population.

## 4. Modern strategies for vaccinating older adults

Despite the fact that in many countries in recent years specific recommendations have been developed for the vaccination of older adults, in global public health there is still no uniform strategy for immunizing this age group of the population and assessing its effectiveness [122]–[127]. One of the problems associated with assessing the effectiveness of vaccination against respiratory infections in the older adults is a significant spread of comorbid conditions, which, combined with aging of immunity, leads to an increase in infectious diseases in this age population [122],[124],[125],[128].

Attention is drawn to the absence in the national recommendations of a unified interpretation of the definition of the category of the population “older adults”. In Poland, this age group is ≥55 years, in some countries (Germany, Greece, Iceland, Slovakia, Russia, etc.) ≥60 years, while in most other EU countries, including France and the UK, ≥65 years [122]–[127].

The results of the few comparative studies of the effectiveness of vaccination against influenza, pneumococcal infection and other infections carried out in Europe and America give an unequivocal answer about a lower antibody titer in older adults compared with younger people [122]–[124],[126],[127]. In this regard, in recent years, there has been an active search for strategies to improve the effectiveness of vaccination, which depends, in particular, on i) the type of vaccines used, the content of antigen, adjuvant, dosage and ii) the schedule of vaccination, the method and place of administration of the vaccine. Of course, the effectiveness of vaccination will depend on the individual characteristics of the patient [127],[129].

In this connection, in order to increase the immunogenicity of drugs in older adults, it is necessary to adapt the available vaccines and immunization schedules for this population category. To this end, for example, to increase the effectiveness of influenza vaccines, various strategies have been used: higher antigen content, alternative delivery routes and the inclusion of adjuvants, which usually led to the appearance of slightly higher antibody titers [123],[125],[126],[128].

One of the problems with assessing the effectiveness of vaccination against respiratory infections in the older adults is a significantly frequent occurrence of comorbid conditions, which, combined with the aging of the immune system, leads to an increase in the incidence of infectious diseases in this age population [123],[128]–[131]. Therefore, it becomes clear that the available COVID-19 vaccines should be adapted for the older adults [124],[130],[132].

Current strategies to improve vaccination effectiveness in the older adults include several approaches that consider the age-related decline in response to immunization. They are based on new knowledge of the immune system's reactions to antigens at the molecular level and a better understanding of the effect of immunosenescence on immunization [122],[128],[133]–[135].

***Strategies for increasing the immunogenicity of available vaccines*.** Over the past decades, health systems in many countries have actively recommended influenza vaccination for older adults. Criticism of these recommendations, associated with the low efficacy of available drugs for this population group, has led to the development of more immunogenic vaccines that provide an optimal magnitude of the immune response.

Strategies for increasing the immunogenicity of existing vaccines proposed in recent years are associated with changing their: i) design features, including the creation of new types, changes in composition, use of antigens, causing a more pronounced immune response, changing the dosage, as well as the inclusion of adjuvants in the formulation; and ii) changes in vaccination regimens (methods and site of administration of drugs, changes in intervals, schedule of immunization) [147]–[152].

For example, an increase in the immunogenicity of vaccines is achieved by the traditional inclusion of special substances in the formulation-adjuvants, such as, aluminum salts, which have been used in this capacity for more than 90 years [123] or modern adjuvant systems: virosomes (lipid nanoparticles), AS04, AS03, Squalene et al. [124],[125],[136]. The main mechanism of virosome action is associated with both the activation of the innate immune response and T- and B-cells initiation [124],[135],[137]. Rational choice of adjuvants in engineered COVID-19 vaccines may provide effective protection for older adults.

In addition, in recent years, fundamentally new strategies for increasing the effectiveness of existing types of vaccines, based on the use of the immunoregulatory functions of the skin and associated administration routes on the safety and immunogenicity of vaccines, have been actively used.

It has been established that the immunogenicity and safety of vaccines largely depends on the route of administration and significantly affects the induction of local and systemic immune responses. Large-scale immunization of the population requires the use of effective, patient-friendly, cost-effective, versatile vaccine delivery methods. The traditional methods of delivery of vaccines are parenteral and transmucosal [138]–[142].

Vaccination of mucous membranes (oral, nasal and other injection sites) is certainly a safer method, which is usually better tolerated by older adults, and the main disadvantage – a relatively weak immune response is solved by the inclusion of safe and effective adjuvants in vaccines [139],[140],[143]–[145]. This method of immunization is supported by a local increase in IgA production, which is not observed with parenteral administration [143],[145]–[147], as well as activation of not only local, but also systemic immune responses [147],[148].

The ongoing massive COVID-19 vaccination indicates that the most common methods of parenteral administration of drugs are intramuscular (IM), intradermal (ID) and subcutaneous (SD) methods using conventional injection needles, despite the established restrictions [147],[149]–[150]. These limitations are related to the dependence of the effectiveness of many traditional types of vaccines on temperature fluctuations, which requires an expensive creation of a cold chain for storing vaccines, which creates additional problems when they are used to immunize the population of underdeveloped countries [148]–[150]. The results of studying the generation of immunogenicity when using parenteral delivery methods using the example of vaccines against hepatitis B, influenza and human papillomavirus, indicate a higher immune response during ID immunization [147],[152],[153].

In addition to traditional methods of delivering vaccines, alternative and innovative methods and devices have been developed in recent years to improve the efficiency and safety of immunization. One of these innovations is the use of various types of microneedles and patches with microchips to administer vaccines (including COVID-19 vaccines) using a microinjector [150],[153]. It has been established that these methods of delivery of drugs targeting the skin are capable of inducing powerful and long-term pathogen-specific protective immunity [150].

The protective role of the skin as a physical barrier is well known and long recognized. However, relatively recently, its unique function as an immune organ was assessed, due to the cutaneous immune mechanisms discovered in recent decades for the initiation and regulation of innate and adaptive immune responses to infectious pathogens [123],[125],[141]–[144],[150].

The attractiveness of the skin for the administration of vaccines is associated with the presence in it of a developed network of capillaries, lymphatic vessels, as well as resident populations of professional immunocompetent cells that initiate an immune response to the antigenic components of vaccines administered through the skin [123],[125],[141],[150]. For example, an open-label randomized trial by Paccalin et al. [143] showed that the immune response to the Intanza vaccine (Sanofi Pasteur, France) against influenza, when administered intradermally to the older adults (60–95 years, N = 3695), proved to be more pronounced compared to intramuscular administration [142]–[144]. In recent years, more and more evidence has emerged in favor of the benefits of intradermal immunization, which is superior to intramuscular and subcutaneous routes of administration [123],[125],[143],[150],[153].

The third strategy to improve vaccines' effectiveness for older adults' patients is to increase the dose or increase the antigen content of influenza-type vaccines [130],[134],[136],[153]. However, despite recent advances in the development of high-dose, adjuvant, and subunit vaccines for immunization against influenza [135],[137],[140], developing more effective vaccines for the older adults, especially those preventing the most severe complications, remains a severe challenge. The need for and timing of revaccination of older adults' people is a subject of ongoing research [139],[141],[146]–[148].

Therefore, the development of vaccination programs and the creation of special and effective COVID-19 vaccines, aimed at the older adults' population, are relevant and timely today [149],[150]–[152]. In this connection, the developed strategies for improving the immune response to antiviral vaccines in the older adults are of particular interest [143]–[145],[150],[153].

All countries have childhood vaccination programs, but few government programs can immunize adults. According to WHO, by 2019, less than 60% of Member States had such influenza vaccination programs. The implementation of child and adult immunization programs differ, first of all, in social expectations, participation of public health, government organizations, and the community, delivery logistics (the need to maintain the “cold chain”), etc.

Thus, more than 40% of states will first face these differences in 2021 when implementing a mass program COVID-19 vaccination, including identifying target populations for priority vaccination and organizing monitoring and evaluating the safety and effectiveness of immunization, including in the older adults.

## 5. Creation of an evidence-based system for assessing the safety and monitoring the effectiveness of COVID-19 vaccines for the older adults (for discussion)

In a situation where different types of vaccines will be used in other countries to immunize the population; it is necessary to pay special attention to developing a unified scientifically based system for assessing the safety and monitoring the effectiveness of COVID-19 vaccines for the older adults. Its development should be at the center of public health attention and should serve the purpose of restoring public confidence in vaccination programs.

**Figure 2.**
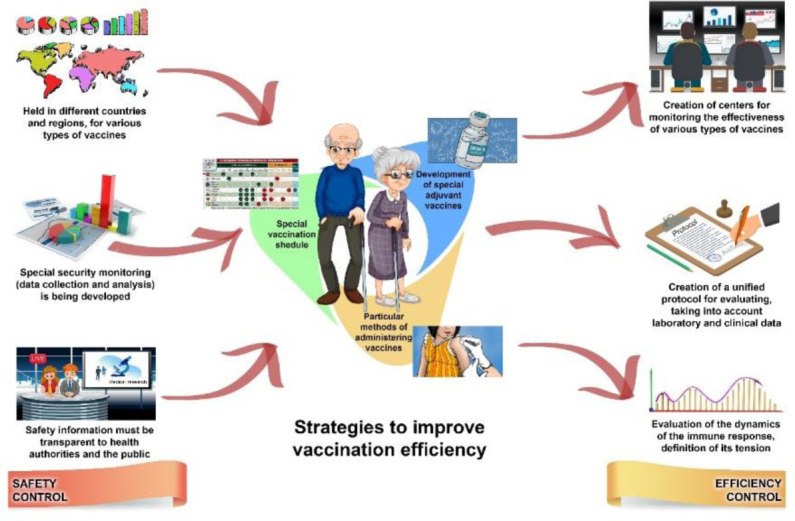
Key strategic directions for monitoring the safety and effectiveness of vaccination in the older adults.

The program should consider the peculiarities of age-related pathophysiological characteristics (aging of the immune system and concomitant diseases) and consist of three directions. These include developing strategies to improve the effectiveness of immunization for the older adults and monitor the safety and efficacy of vaccination for this population (Figure 2).

***Strategies for improving vaccination design*.** These strategies will differ between the types of vaccine used (adaptation or increase in the immunogenicity of existing vaccines, change in the vaccination schedule, change in the method of administration of drugs, etc.). All the proposed strategies require substantiation and confirmation of their effectiveness in a clinical setting. Furthermore, an essential factor that provides success of vaccination is the rational decision on its priority for particular social and age groups.

***Vaccination sequence*.** Recommendations on the vaccination sequence are published on the WHO and SDS websites. However, due to the increasingly spreading pandemic, each country's government makes its own decision on the priority of immunization. In the context of the problem, it should be taken into account that, in addition to occupational risk groups (hundreds of thousands), there are also social age risk groups (dozens and hundreds of millions) of older people. This category of the population, for the most part, determines the current level of morbidity and mortality from COVID-19, while sick, older adults' people themselves are the focus of infection. Therefore, older people aged 65+ should be included in priority groups (after medical and main first-line staff).

***Vaccination safety control*.** Vaccine safety is a vital component of an immunization campaign for the general population and, especially, for older people. Based on international experience in organizing such events, the safety control strategy should consist of three principles: (i) multicenter safety control of various types of vaccines; (ii) development of a special monitoring system, including active collection and analysis of data on side effects of vaccination for selected age groups of the population with an open and accessible database and readiness of healthcare authorities for emergencies; and (iii) availability of information on monitoring results for the healthcare institutions and the public.

The participation of the public and medical practitioners (except for experts from public healthcare institutions and universities) in discussing the organization of COVID-19 vaccination, its need, risks, and complications is essential. This is a necessary and one of the critical factors of the success of the entire immunization campaign and a factor that provides increasing public confidence in vaccines.

Due to the use of different types of vaccines in different countries, objective information on side effects, complications, and deaths should be accumulated on the websites of WHO, SDS, and national healthcare ministries in statistical overviews and analytical reports and regularly updated.

In addition, COVID-19 vaccination should also be recommended as an integral part of the prevention of cardiovascular disease in older adults. This policy is based on the results of clinical observations showing a high incidence of cardiovascular damage in patients with COVID-19, especially relevant for the age group of the population [154],[155].

***Monitoring the effectiveness of vaccinations*.** One of the results of the international European projects' effort on monitoring the effectiveness of existing influenza and pneumococcal vaccines (I-MOVE-plus and SpIDnet) and vaccination strategies for the older adults was the conclusion that this type of monitoring is necessary [156],[157].

Monitoring the effectiveness of vaccinations should include as follows [158]:

(a) control measures for certain age groups of older adults' people (65–75 years; 75–80 years; >80 years) depending on the initial level of health and the concomitant diseases revealed;

(b) assessment of the initial level of indicators of the immune status in the older adults of the selected groups;

(с) evaluation of the effect of vaccines on blood biochemical parameters (activity of lactate dehydrogenase, albumin, C-reactive protein, D-dimer, potassium, high-density lipoprotein cholesterol and procalcitonin) in the observation groups; comparative analysis of the reliability of the results in different groups and comparison with the original data;

(d) analysis of seasonal dynamics of the immune response to vaccines in the older adults' population (specific antibodies IgM and IgG), which, obviously, will vary depending on the region, race, gender, or type of vaccines used.

It should be borne in mind that the appearance of antibodies in the blood serum may not be the only indicator of protection and, accordingly, evidence of the effectiveness of immunization. Although specific T-cell immune responses in older adults are impaired, the recruitment of cytotoxic T-lymphocytes, which purge virus-infected human cells and provide control of the infection process, may serve as a more significant indicator of protection against COVID-19 than seroconversion [160],[161].

(e) determination of the level of post-vaccination immunity for the vaccines used in all the age cohorts and assessment of the significance of differences, which will allow a conclusion as to whether the response to the vaccine is dependent on baseline health.

With a larger sample size and multicenter studies, these interventions can provide assessment of the vaccination effectiveness.

## 6. Conclusions

Vaccination is a crucial public healthcare strategy in communicable disease control. The world practice of immunization of the population made it possible to assess its problems and reduce morbidity and mortality from bacterial and viral infections among the general population. The hope for successfully eliminating the current COVID-19 pandemic is associated with creating new and effective vaccines.

The success of the growing mass vaccination of the population of many countries against the new coronavirus infection depends on the degree of public health priority and attention to this age group of the population. In this context, the relative lack of research and scant evidence of the safety and efficacy of the proposed COVID-19 vaccines for older adults require special attention, due to the unprecedented timing of their development.

The current global health crisis associated with the rapid spread and destructive nature of the current pandemic is associated with a crisis in the attitude of society towards older adults, whose number in the near future will amount to up to a quarter of the world's population. Modern biotech companies have learned to manufacture various types of vaccines and therapeutics for treating bacterial and viral infections.

However, over the past decades, the age category of the “older adults” population has been systematically excluded from clinical trials of therapeutic and prophylactic drugs under the pretext of multimorbidity, increased susceptibility to side effects, and decreased functionality of organs and systems [162]. Since the presence of “competing risks” could call into question the effectiveness and safety of the tested medicinal and prophylactic agents.

The figurative etymology of the Chinese character “weiji” (“crisis”) interprets its double meaning as “danger” and “opportunity”. Of course, there is a “danger” in the problem of the growing COVID-19 pandemic. It is related to the current statistics (April 2021) of the number of cases (>136 million) and deaths (>2.9 million), which is alarming and does not yet show a downward trend.

However, behind the optimistic reports about the speed of development, testing and introduction of various types of national vaccines, there are old habits of biotech firms and campaigns to ignore the interests of older adults, who should benefit most from the safety and effectiveness of vaccinations. The authors would like to believe that the second meaning of the hieroglyph—“opportunity” was realized by society on the example of the current pandemic and the development of a special strategy for creating vaccines, immunization schemes and monitoring its effectiveness for the age groups of the population 60+, 70+, 80+, etc. taking into account the physiological aging processes of the body systems.

Overcoming the public mistrust in vaccination programs against the new coronavirus infection is possible, among other things, with a stable and scientifically based system created for assessing the safety and monitoring the effectiveness of COVID-19 vaccines, primarily for the older adults. The apparent necessity to create such an approach has arisen from the society's need for objective data to make reasoned decisions about the optimal type of vaccine and effective prevention strategies, regardless of commercial interest and political ambitions.
